# Gearbox Fault Diagnosis Under Noise and Variable Operating Conditions Using Multiscale Depthwise Separable Convolution and Bidirectional Gated Recurrent Unit with a Squeeze-and-Excitation Attention Mechanism

**DOI:** 10.3390/s25102978

**Published:** 2025-05-08

**Authors:** Xiaoteng Ma, Kejia Zhai, Nana Luo, Yehui Zhao, Guangming Wang

**Affiliations:** 1 College of Information Science and Engineering, Shandong Agricultural University, Taian 271018, China; maxiaoteng2025@163.com (X.M.); 2024121182@sdau.edu.cn (K.Z.); 2 College of Mechanical and Electronic Engineering, Shandong Agricultural University, Taian 271018, China; lnn323050@163.com; 3 College of Engineering, Ocean University of China, Qingdao 266404, China; zhaoyehui963@163.com; 4 National Engineering Research Center of Agricultural Production Machinery and Equipment, Taian 271018, China

**Keywords:** gearbox, fault diagnosis, convolutional neural network, gated recurrent unit, attention mechanism

## Abstract

Gearbox condition monitoring is essential for ensuring the reliability of power transmission systems. However, the existing methods are constrained by shallow feature extraction and unidirectional temporal modeling. To address these limitations, this study proposes a novel fault diagnosis framework that integrates multiscale depthwise separable convolution, bidirectional gated recurrent units, and a squeeze-and-excitation attention mechanism. This approach enables multiscale feature extraction from vibration signals, bidirectional temporal modeling, and the enhancement of critical fault-related information. The experimental results demonstrate that the proposed method significantly outperforms conventional models in terms of fault diagnosis accuracy, noise robustness, and adaptability to varying operating conditions. The attention mechanism effectively suppresses noise interference, while bidirectional temporal modeling accurately captures fault propagation characteristics, thereby improving adaptability to dynamic conditions. This research provides a highly robust solution for intelligent gearbox fault diagnosis in complex industrial environments.

## 1. Introduction

Gearboxes enable power and load matching by changing the transmission ratio and are extensively utilized across various fields such as vehicles, industry, and energy. Their health status directly affects the reliability, safety, and economic performance of equipment. However, due to prolonged exposure to complex working conditions—such as high loads, high-speed operation, and temperature fluctuations—critical internal components, including gears and bearings, are highly susceptible to wear, cracks, tooth breakage, cage fracture, and thermal damage, among others [1,2,3,4,5]. These faults can result in equipment shutdowns, system failures, and even major safety incidents. Therefore, research on gearbox fault diagnosis is of significant theoretical and engineering value.

Traditional gearbox fault diagnosis methods primarily rely on techniques such as vibration signal analysis, noise analysis, oil debris analysis, power analysis, and temperature monitoring. Among them, vibration signal analysis is widely adopted due to its physical correlation, high sensitivity, rich feature extraction methods, and fast dynamic response. Common vibration signal processing techniques include time-domain, frequency-domain, and time–frequency analysis, e.g., Empirical Mode Decomposition (EMD) [6], wavelet transform (WT) [7], short-time Fourier transform (STFT) [8], and Fast Fourier Transform (FFT) [9]. For instance, Wang et al. [10] combined EMD with improved variational mode decomposition to identify nonlinear and non-stationary pitting-wear signals in gearbox gears. Among them, EMD is used for noise reduction to highlight characteristic frequencies. Huang et al. [11] proposed an adaptive short-time Fourier transform based on STFT and combined it with the synchroextracting transform to diagnose gearbox faults under time-varying speeds. Strömbergsson et al. [12] conducted a comparative study on the application of FFT and WT in the fault diagnosis of wind turbine gearbox bearings.

In the past decade, machine learning technology has also been widely applied in fault diagnosis. Compared to the above signal processing methods, machine learning is data-driven, does not rely on expert knowledge, and offers greater adaptability and portability. For instance, Hou et al. [13] proposed an entropy-based method called multivariate multiscale cross-fuzzy entropy and combined it with a Support Vector Machine (SVM) for gearbox fault diagnosis. Felix et al. [14] introduced a data augmentation and balancing method based on sparse autoencoder technology and used SVM and Random Forest (RF) classifiers to detect gearbox faults more effectively. Bao et al. [15] developed an active learning algorithm that integrates multiple strategies to reduce sample datasets, thereby enhancing the efficiency of KNN in gearbox fault diagnosis. In addition to these standalone applications, machine learning can also be used in conjunction with signal processing techniques to improve algorithm performance, as detailed in references [16,17,18].

Deep learning methods, such as Convolutional Neural Networks (CNNs) and Recurrent Neural Networks (RNNs), are also data-driven but do not require manual feature extraction like signal processing and traditional machine learning, thereby greatly expanding their application potential. CNNs can accept 1D vibration signals as input for gearbox fault diagnosis, as detailed in Huang et al.’s [19] research, although they are better suited for processing image data. To address this limitation, researchers have developed various methods to convert vibration signals into images. For instance, Wang et al. [20] converted vibration signals into snowflake images using the Symmetrized Dot Pattern (SDP) method, while Pang et al. [21] achieved this conversion through Bispectrum (BSP) analysis. Similar approaches can be found in the work of Mao et al. [22]. However, this preprocessing step involving image conversion occurs not only during training but also during deployment, which significantly reduces the real-time performance of the algorithm. In contrast, RNNs and their variants, such as Long Short-Term Memory (LSTM) and gated recurrent units (GRUs), are adopted for processing time-series data and capturing dynamic signal features. In this field, Andhale et al. [23] combined opposition learning with the artificial humming-based crow search algorithm to optimize RNN weights for gearbox fault diagnosis. Chen et al. [24] proposed an improved LSTM model called Speed-Integrated LSTM (SI-LSTM), which demonstrates strong adaptability to non-stationary gearbox fault signals. Su et al. [25] introduced a Normal Behavior Modeling (NBM) method that integrates a spatial–temporal attention module and GRU for gearbox fault diagnosis. In recent years, hybrid models that couple CNN and RNN architectures have demonstrated improved performance by combining spatial feature extraction with the effective modeling of long-term dependencies in time-series data. These approaches have shown significant advantages in handling non-stationary signals, complex fault modes, and high-noise environments. For example, Chen et al. [26] developed a CNN-GRU model based on multi-sensor signals for gearbox fault diagnosis. Li et al. [27] applied the Gray Wolf Optimization (GWO) algorithm to fine-tune hyperparameters in a 1DCNN-GRU network for enhanced feature learning. Yin et al. [28] introduced a composite fault diagnosis model for rolling bearings, incorporating an enhanced dual-channel Deep Residual Shrinkage Network (DRSN) and GRU structure to mitigate the impact of noisy signals. Han et al. [29] used CNN-LSTM to address the problems of sample imbalance and noise interference in gearbox fault diagnosis. Additionally, the introduction of attention mechanisms can enhance the extraction of key features while suppressing irrelevant ones. For example, Wang et al. [30] proposed a novel fault diagnosis method that fuses a Squeeze-and-Excitation Multiscale Convolutional Neural Network (SENetMSCNN) with GRU to address the problem of low diagnostic accuracy caused by an overrepresentation of normal samples in large-scale vibration data. Liu et al. [31] embedded squeeze-and-excitation channel attention mechanisms into the ResNet34 network and developed a SE-ResNet model that demonstrates strong feature extraction capabilities in high-noise environments; Shao et al. [32] designed a Residual-level Discrete Wavelet Convolution (RDC) block based on the discrete wavelet transform and squeeze-and-excitation (SE) attention mechanism, and constructed a spatiotemporal fusion deep feature extraction network (RDC-BIGRU) based on it. This network can filter out high-frequency interference components and enhance the temporal representation of features.

In practical industrial applications, the vibration signals generated by gearboxes often exhibit multiscale, non-stationary, and nonlinear characteristics. Although traditional CNNs can effectively capture local spatial features, their ability to model long-term dependencies is limited, which makes it difficult to extract multiscale features efficiently [33]. In addition, while CNNs can automatically extract vibration signal features, they lack the ability to filter out irrelevant features or emphasize the most critical ones. If the extracted features are not representative, the accuracy of fault diagnosis may be compromised [34]. RNNs and their variants suffer from the vanishing gradient problem when processing long-sequence data, which leads to limited modeling capacity and high computational complexity in fault diagnosis. Multiscale convolution aggregates features from multiple receptive fields by embedding multiscale information, thereby improving CNN performance in the spatial dimension [35]. In addition to algorithmic improvements, it is essential to consider specific real-world application scenarios when developing gearbox fault diagnosis models, as discussed in [36,37,38,39]. However, current research addressing gearbox fault diagnosis under complex conditions—particularly those involving high noise levels and variable operating environments—remains limited.

Therefore, to address the above issues, this paper proposes a novel fault diagnosis model, MDSC-SE-BiGRU, which combines multiscale depthwise separable convolution, bidirectional gated recurrent units, and a squeeze-and-excitation attention mechanism. The main innovations and contributions are as follows:

(1) Multiscale feature extraction: The introduction of multiscale depthwise separable convolution enables the efficient extraction of multiscale spatial features from vibration signals through multiscale convolutional kernels, significantly enhancing feature extraction capabilities.
(2) Adaptive feature recalibration: The incorporation of the squeeze-and-excitation attention mechanism dynamically adjusts channel weights, enhancing the model’s focus on critical fault features, and thus promoting the ability of multiscale convolution to capture key information.
(3) Bidirectional temporal modeling: The bidirectional gated recurrent unit is employed to capture temporal dependencies in both directions, allowing the model to simultaneously consider past and future contextual information, thereby improving its sequential modeling ability.
(4) Efficient computation and high diagnostic accuracy: By introducing depthwise separable convolutions, computational complexity is effectively reduced, significantly improving efficiency while maintaining high performance. Moreover, the integration of multiscale feature extraction and time-dependent modeling has significantly improved diagnostic accuracy without compromising efficiency.

Based on these innovations, the proposed MDSC-SE-BiGRU model demonstrates enhanced robustness and generalization performance compared to the existing methods. It effectively addresses the challenges of low diagnostic accuracy in gearboxes under noise interference and variable operating conditions.

The structure of this paper is organized as follows: Section 1 introduces the research background of gearbox fault diagnosis, the limitations of the existing methods, and the objectives of this study. Section 2 introduces the fundamental theories of multiscale convolutional networks, temporal dependency modeling, and squeeze-and-excitation. Section 3 describes the MDSC-SE-BiGRU model, including its theoretical basis and structural design. Section 4 evaluates the effectiveness of the proposed model through experiments conducted on two datasets. By comparing the model with the existing approaches, its superior performance in gearbox fault diagnosis is validated. Section 5 summarizes the key findings and discusses potential directions for future research.

## 2. Related Theoretical Background

In recent years, deep learning techniques have made substantial advances in mechanical fault diagnosis, particularly with the application of Multiscale Convolutional Neural Networks (MSCNNs) and gated recurrent units (GRUs). MSCNN is widely employed for diagnosing vibration signals and multi-sensor data due to its ability to effectively process complex, non-stationary signals through multiscale feature extraction. GRU excels in handling time-series data and performs remarkably well on tasks with time-dependent characteristics.

### 2.1. Multiscale Convolutional Neural Network (MSCNN)

**Figure 1:**
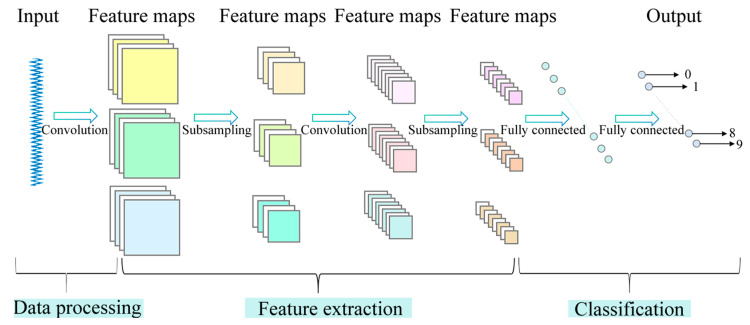
Structure of MSCNN.

The Multiscale Convolutional Neural Network (MSCNN) is an advanced deep learning architecture designed to enhance the feature extraction capabilities of traditional Convolutional Neural Networks (CNNs), particularly in applications involving non-stationary and multi-frequency signals such as vibration data in fault diagnosis. Unlike conventional CNNs that operate at a single scale, MSCNN incorporates multiple parallel convolutional branches with diverse kernel sizes to extract multiscale feature representations. This design allows the model to simultaneously capture both fine-grained and coarse-grained information from the signal, resulting in a more comprehensive representation of fault characteristics. The structural framework of MSCNN is illustrated in Figure 1.

The design of MSCNN is primarily based on the following fundamental mathematical formulations and principles:

(a) Convolutional Layer: Suppose the input signal is X, and the convolution kernel is W. The convolution operation is defined as follows:
  (1) $$Y=f\left(X*W+b\right)$$
  where f is the activation function, b is the bias term, and ∗ represents the convolution operation. The resulting output feature map is denoted as Y.
(b) Pooling Layer: Pooling operations are used to reduce dimensionality and extract essential features. Common pooling methods include Max Pooling and average pooling. The output of the pooling layer can be expressed as follows:
  (2) $$Y_{pooled}=max\left(X_{i}\right)$$
  where $X_{i}$ represents the elements within the pooling window, and max represents the maximum pooling operation.
(c) Multiscale Convolution: A key innovation of MSCNN is its use of multiscale convolutional operations. Suppose there are multiple convolution kernels $W_{1},W_{2},\ldots ,W_{n}$, each with a different size. These kernels extract features at various scales from the input signal. The final output feature $Y_{final}$ is the fusion of convolution feature maps at multiple scales:
  (3) $$Y_{final}=\sum_{i=1}^{n}f\left(X*W_{i}+b_{i}\right)$$
  where $Y_{final}$ represents the final multiscale feature maps extracted by the MSCNN model.

This multiscale architecture ensures that features extracted from various frequency bands are preserved and integrated, which makes MSCNN highly suitable for tasks such as fault detection in mechanical systems.

### 2.2. Gated Recurrent Unit (GRU)

**Figure 2:**
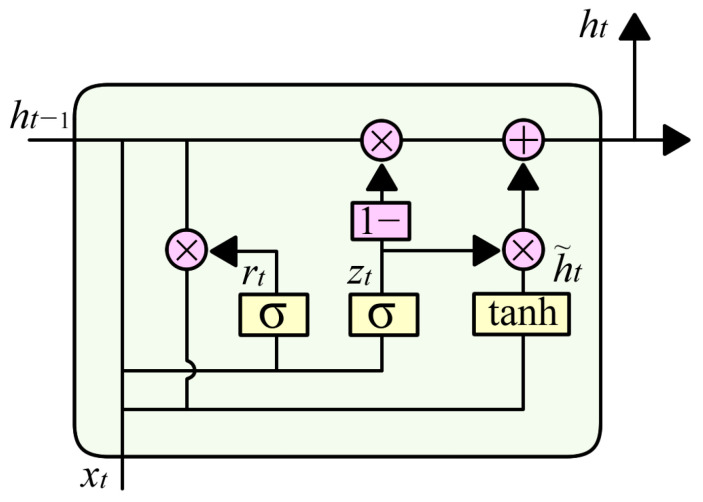
Structure of GRU.

The gated recurrent unit (GRU) is a refined variant of the Recurrent Neural Network (RNN) specifically designed for modeling sequential dependencies in time-series data. By introducing gating mechanisms that regulate the flow of information, GRU effectively mitigates the vanishing gradient problem inherent in traditional RNNs. Its structure is particularly effective for capturing long-range temporal dependencies in vibration signals associated with mechanical faults. The structure of the GRU model is depicted in Figure 2.

The GRU model consists of the following core components, and the forward propagation process can be mathematically formulated as follows:

(a) Update Gate: The update gate $z_{t}$ controls the degree to which the current state is updated. It is computed as follows:
  (4) $$z_{t}=\sigma \left(W_{z}x_{t}+U_{z}h_{t-1}+b_{z}\right)$$
  where $x_{t}$ represents the input at time step *t*, $h_{t-1}$ is the previous hidden state, σ is the Sigmoid activation function, and $W_{z}$ and $b_{z}$ are learnable parameters.
(b) Reset Gate: The reset gate $r_{t}$ determines how much of the past hidden state contributes to the current state. It is defined as follows:
  (5) $$r_{t}=\sigma \left(W_{r}x_{t}+U_{r}h_{t-1}+b_{r}\right)$$
  where $W_{r}$ and $b_{r}$ are the parameter matrices and bias vectors for the reset gate, σ represents the sigmoid activation function, which bounds the gate values between 0 and 1, $x_{t}$ is the input vector at time step t, $h_{t-1}$ is the hidden state from the previous time step;
(c) Candidate Hidden State: The candidate hidden state ${\tilde{h}}_{t}$ is computed using the reset gate to determine the current state information:
  (6) $${\tilde{h}}_{t}=tanh\left(W_{h}x_{t}+U_{h}\left(r_{t}\cdot h_{t-1}\right)+b_{h}\right)$$
  where ${(r}_{t}\cdot h_{t-1})$ indicates that the hidden state from the previous time step, $h_{t-1}$ is combined with the reset gate $r_{t}$ to control its influence, $W_{h}\;and\;{\;b}_{h}$ are the corresponding weight matrices and biases, and tanh is the hyperbolic tangent activation function.
(d) Final Hidden State: The final hidden state $h_{t}$ is obtained as a weighted combination of the previous hidden state and the candidate hidden state:
  (7) $$h_{t}=\left(1-z_{t}\right)\cdot h_{t-1}+z_{t}\cdot {\tilde{h}}_{t}$$
  where $z_{t}$ is the update gate, $h_{t-1}$ is the previous hidden state, ${\tilde{h}}_{t}$ is the candidate hidden state, $\left(1-z_{t}\right)\cdot h_{t-1}$ represents retained historical information, and $z_{t}\cdot {\tilde{h}}_{t}$ represents newly acquired information.

The GRU architecture utilizes two fundamental types of weight matrices that govern its temporal dynamics:

(1) Input-to-hidden matrices, which transform the input vector $x_{t}$ into the respective internal gate and candidate state spaces:
  (a) $W_{z}\in R^{n_{h}\times n_{x}}$ connects to the update gate and maps the input vector $x_{t}\in R^{n_{x}}$ to the update gate $z_{t}\in R^{n_{h}}$.
  (b) $W_{r}\in R^{n_{h}\times n_{x}}$ connects to the reset gate and maps the input vector $x_{t}$ to the reset gate $r_{t}\in R^{n_{h}}$.
  (c) $W_{h}\in R^{n_{h}\times n_{x}}$ connects to the candidate hidden state and maps the input vector $x_{t}$ to the candidate hidden state ${\tilde{h}}_{t}\in R^{n_{h}}$.
(2) Hidden-to-hidden (recurrent) matrices, which transform the previous hidden state $h_{t-1}$ to each corresponding gate:
  (a) $U_{z}\in R^{n_{h}\times n_{h}}$ recurrent weights for the update gate; maps the hidden state $h_{t-1}\in R^{n_{h}}$ to the update gate $z_{t}$.
  (b) $U_{r}\in R^{n_{h}\times n_{h}}$ recurrent weights for the reset gate; maps $h_{t-1}$ to the reset gate $r_{t}$.
  (c) $U_{h}\in R^{n_{h}\times n_{h}}$ recurrent weights for the candidate hidden state; maps $h_{t-1}$ to the candidate hidden state ${\tilde{h}}_{t}$.

Where $n_{x}$ represents the dimensionality of the input vector $x_{t}$, and $n_{h}$ represents the dimensionality of the hidden state $h_{t}$. Together, these six weight matrices govern the nonlinear transformations that modulate the memory update and reset mechanisms within the GRU cell. Bias terms $b_{z}$, $b_{r}$, and $b_{h}$, each of dimension $n_{h}$, are included to improve model flexibility by introducing learnable offsets into the respective affine transformations.

### 2.3. Squeeze-and-Excitation Attention Mechanism (SE)

The squeeze-and-excitation (SE) attention mechanism is a pivotal module in the MDSC-SE-BiGRU model, designed to adaptively adjust the weights of channel features. By dynamically emphasizing critical information and suppressing irrelevant or noisy features, the SE module significantly enhances diagnostic performance in complex environments. The SE module comprises two steps—squeeze and excitation—which utilize global average pooling (GAP) and fully connected (FC) layers to generate channel attention weights. These weights are then applied to the original feature maps, thereby achieving adaptive feature enhancement.

In the squeeze step, the SE module compresses the spatial information in each channel into a single scalar by applying global average pooling, thereby generating channel descriptors. Given an input feature map of the shape $\left[C,H,W\right]$, where C represents the number of channels, and H and W denote the height and width of the feature map, respectively, the global average pooling operation is defined as follows:

(8) $$z_{c}=\frac{1}{H\times W}\sum_{i=1}^{H}\sum_{j=1}^{W}x_{c}\left(i,j\right)$$

where $x_{c}\left(i,j\right)$ represents the feature value of the *c*-th channel at the spatial location $\left(i,j\right)$, and $z_{c}$ is the corresponding channel descriptor. By compressing the spatial dimensions, this step extracts global statistical information across different channels.

In the excitation step, the SE module generates channel attention weights through two fully connected (FC) layers. The first FC layer reduces the dimensionality of the channel descriptors $z_{c}$ from C to C/r (where r is the reduction ratio), introducing nonlinearity via the ReLU activation function. The second FC layer restores the dimensionality to C and applies the Sigmoid activation function to produce the channelwise attention weights $s_{c}$. The formulation is defined as follows:

(9) $$s_{c}=\sigma \left(W_{2}\cdot \delta \left(W_{1}\cdot z_{c}\right)\right)$$

where $W_{1}$ and $W_{2}$ are the weight matrices of the two FC layers. δ denotes the ReLU activation function. σ represents the Sigmoid activation function and $s_{c}$ is the resulting channel attention weight vector. Finally, the computed channel attention weights $s_{c}$ are applied to the original feature map via elementwise multiplication, producing the following refined feature map:

(10) $${\tilde{x}}_{c}=s_{c}\cdot x_{c}$$

This adaptive weighting mechanism enables the model to focus on critical features essential for fault diagnosis while suppressing noise and irrelevant information.

The SE module plays a crucial role in enhancing the MDSC-SE-BiGRU model by providing the following benefits: firstly, by adaptively adjusting the weights of channel features, the SE module significantly improves the model’s sensitivity to important fault-related features, leading to higher classification performance. Moreover, the SE mechanism effectively suppresses noise and redundant information, strengthening the model’s stability in complex industrial environments. Lastly, the visualization of channel attention weights offers insight into the model’s decision-making process, thereby enhancing interpretability. By incorporating the squeeze-and-excitation attention mechanism, the SE module provides efficient feature enhancement capabilities, further improving the accuracy and reliability of fault diagnosis in the MDSC-SE-BiGRU model.

### 2.4. Multiscale Depthwise Separable Convolution (MDSC)

**Figure 3:**
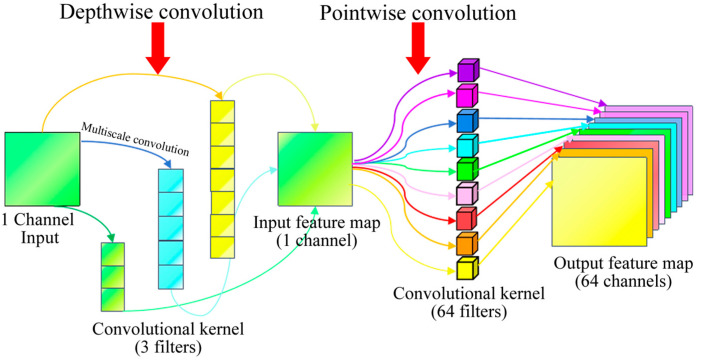
Architecture of MDSC module.

The multiscale depthwise separable convolution (MDSC) module serves as the core component of the MDSC-SE-BiGRU model. It is designed to efficiently extract both low-frequency and high-frequency features from the input signal using multiscale convolution kernels while significantly reducing computational complexity. MDSC adopts a depthwise separable convolution structure, which decomposes the standard convolution into two sequential steps: depthwise convolution and pointwise convolution. In the depthwise convolution, a separate filter is applied to each input channel, generating feature maps with the same number of channels as the input. The convolution kernel has a shape of $\left[C_{in},K,K\right]$, where K represents the kernel size. The number of parameters is $C_{in}\times K\times K$. Subsequently, the pointwise convolution applies a 1 × 1 convolution kernel to map the number of output channels for deep convolution from $C_{in}$ to the target number of output channel $C_{out}$, with a parameter count of $C_{in}\times C_{out}$. The total number of parameters in a depthwise separable convolution is $C_{in}\times K\times K+C_{in}\times C_{out}$. Compared to the parameter count of standard convolution: $C_{in}\times C_{out}\times K\times K$, the number of parameters and computational complexity are significantly reduced. The structure of the MDSC module is shown in Figure 3.

To further enhance feature extraction, the MDSC module employs three parallel convolutional kernels with different sizes (3 × 1, 5 × 1, and 7 × 1) to capture multiscale features from the signal. These kernels extract both local details and global trends to ensure a comprehensive feature representation. Specifically, the following features are captured:

(a) The 3 × 1 convolution captures fine-grained local structures:
  (11) $$x_{1}=DepthwiseSeparableConv\left(x,kernel\_size=3,padding=1\right)$$
(b) The 5 × 1 convolution extracts mid-range contextual dependencies:
  (12) $$x_{2}=DepthwiseSeparableConv\left(x,kernel\_size=5,padding=2\right)$$
(c) The 7 × 1 convolution focuses on broader global trends:
  (13) $$x_{3}=DepthwiseSeparableConv\left(x,kernel\_size=7,padding=3\right)$$

The outputs of these three convolutional pathways are concatenated to form a unified multiscale feature representation, mathematically expressed as follows:

(14) $$x=Concat\left(x_{1},x_{2},x_{3}\right)$$

where $x_{1},x_{2},\;and\;x_{3}$ correspond to the output features from the 3 × 1, 5 × 1, and 7 × 1 convolutional pathways, respectively. This multiscale feature extraction strategy enables the model to simultaneously capture both low-frequency and high-frequency components of the signal, which enhances its ability to perceive complex fault signals.

The MDSC module is designed to achieve the following objectives: Firstly, by leveraging depthwise separable convolutions, MDSC significantly reduces parameter count and computational complexity, making it well suited for deployment in resource-constrained environments. Secondly, the use of multiple convolutional kernels enables the simultaneous extraction of both low-frequency and high-frequency features from the signal, thereby improving the model’s ability to characterize complex fault signals. Finally, the fusion of multiscale features improves the model’s generalization ability, allowing it to effectively adapt to fault signals with varying frequency components. By combining multiscale feature extraction with depthwise separable convolution, the MDSC module provides a highly efficient and comprehensive feature input for subsequent temporal sequence modeling and feature enhancement, thereby establishing a solid foundation for gearbox fault diagnosis.

### 2.5. Bidirectional Gated Recurrent Unit (BiGRU)

**Figure 4:**
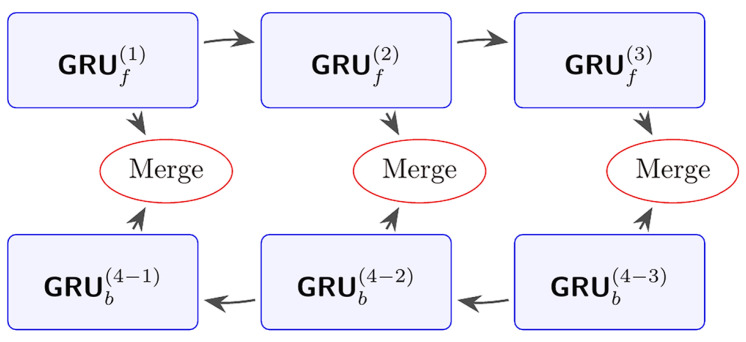
Architecture of BiGRU model.

As an extension of the standard GRU introduced previously, the bidirectional gated recurrent unit (BiGRU) plays a crucial role in the MDSC-SE-BiGRU model, which is specifically designed to capture bidirectional temporal dependencies in time-series fault signals. By utilizing two parallel GRU layers—one that processes the sequence forward and the other backward—BiGRU constructs a more comprehensive temporal representation, which is especially effective for analyzing complex mechanical vibration signals. The structure of BiGRU is illustrated in Figure 4.

The output of BiGRU is obtained by concatenating the outputs from both the forward and backward GRU layers, mathematically expressed as follows:

(a) Forward GRU

The forward GRU processes the time-series data sequentially from the past to the future, capturing temporal dependencies in the forward direction. At each time step *t*, the hidden state $h_{t}^{forward}$ is updated based on the current input $x_{t}$ and the previous hidden state $h_{t-1}^{forward}$. The forward GRU cell ${GRU}_{forward}$ incorporates gating mechanisms, including the update gate, reset gate, and candidate hidden state, which collectively govern the flow of information. This is formulated as follows:

(15) $$h_{t}^{forward}={GRU}_{forward}\left(x_{t},h_{t-1}^{forward}\right)$$

(b) Backward GRU

The backward GRU processes the time-series data in reverse order, from the future to the past, thereby capturing temporal dependencies in the backward direction. At each time step *t*, the hidden state $h_{t}^{backward}$ is updated based on the current input $x_{t}$ and the subsequent hidden state $h_{t+1}^{backward}$. The backward GRU cell ${GRU}_{backward}$ is structurally analogous to the forward GRU cell but operates in the reverse temporal direction, effectively complementing the forward processing path. This is formulated as follows:

(16) $$h_{t}^{backward}={GRU}_{backward}\left(x_{t},h_{t+1}^{backward}\right)$$

(c) Output Fusion

The outputs from the forward and backward GRU layers are concatenated at each time step to form the final hidden state $h_{t}$:

(17) $$h_{t}=\left[h_{t}^{forward},h_{t}^{backward}\right]$$

This concatenation operation ensures that each time step’s hidden state contains information from both past-to-future and future-to-past sequences, thereby enabling the model to obtain a more comprehensive understanding of the time-series data.

By incorporating contextual information from both directions, the BiGRU module enhances the model’s ability to extract meaningful temporal features. This integration not only improves robustness against noise and data variability but also significantly strengthens the diagnostic performance of the MDSC-SE-BiGRU model.

## 3. MDSC-SE-BiGRU Model

**Figure 5:**
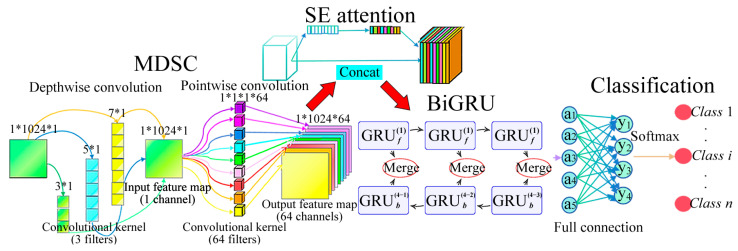
Network structure of MDSC-SE-BiGRU model.

This section introduces an innovative deep learning architecture, MDSC-SE-BiGRU, which integrates multiscale depthwise separable convolution (MDSC), the squeeze-and-excitation (SE) attention mechanism, and bidirectional gated recurrent unit (BiGRU). This hybrid model enables a synergistic optimization of multiscale feature extraction, temporal sequence modeling, and adaptive feature selection for vibration signals. The architecture of the MDSC-SE-BiGRU model is illustrated in Figure 5.

First, the MDSC module employs three parallel convolutional kernels with different sizes (3 × 1, 5 × 1, and 7 × 1) to extract multiscale features from the input signal. The depthwise separable convolution structure significantly reduces computational complexity while effectively capturing both fine-grained local details and global contextual trends in the signal. Second, the SE module applies channelwise attention weighting to the multiscale features extracted by the MDSC module. It first uses global average pooling to extract statistical dependencies between channels, and then generates channelwise importance weights through a fully connected layer. This mechanism dynamically enhances crucial fault features while suppressing irrelevant or redundant information, thereby improving the feature representation capability. Third, the BiGRU module processes the features enhanced by SE for bidirectional temporal sequence modeling. A two-layer BiGRU is employed to simultaneously capture both forward and backward temporal dependencies, comprehensively reflecting the dynamic evolution of fault signals. Finally, the fully connected layer maps the output from the BiGRU to the corresponding fault class space, and the Softmax activation function is then applied to generate the probability distribution over fault categories, thereby completing the fault diagnosis task. The network model is built under the framework of PyTorch 2.4.1 based on Python 3.8. The concrete network parameters of the MDSC-SE-BiGRU model are shown in Table 1.

## 4. Results and Discussion

### 4.1. Case 1: Fault Diagnosis of Planetary Gearbox

#### 4.1.1. Data Description

**Figure 6:**
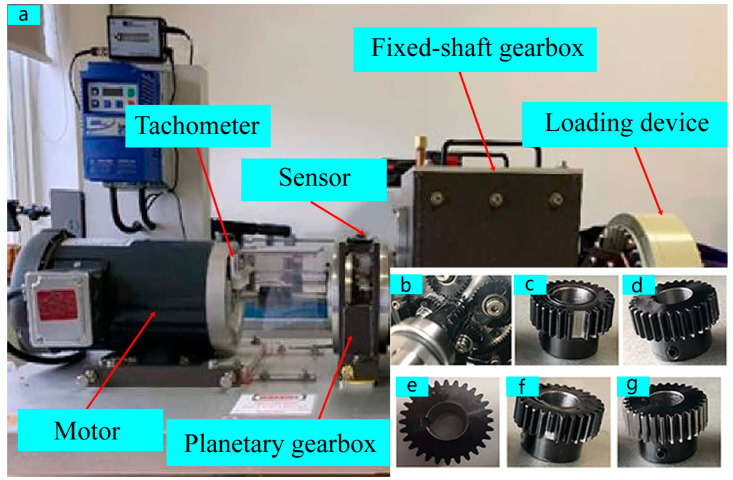
Test bench for Case 1: (**a**) structure of test bench; (**b**) internal configuration of planetary gearboxes; (**c**) missing tooth; (**d**) cracked tooth; (**e**) healthy condition; (**f**) broken tooth; (**g**) worn tooth.

Case 1 is from the WT-Planetary gearbox dataset [40], which contains vibration signals collected from the input shaft of the planetary gearbox in both X and Y directions, as well as encoder data, consisting of five distinct health states (broken tooth, healthy, missing tooth, cracked tooth, and worn tooth) and eight rotational speed settings (20 Hz, 25 Hz, 30 Hz, 35 Hz, 40 Hz, 45 Hz, 50 Hz, and 55 Hz). As shown in Figure 6a, the test bench consists of a motor, planetary gearbox, fixed-shaft gearbox, and loading device. The failure of the system originates from the sun gear of the planetary gearbox, as shown in Figure 6b, and the five health states are shown in Figure 6c–g. The dataset used in this study offers more than five minutes of continuous monitoring data for each condition of the gearbox. This substantial sample volume offers robust support for training and optimizing deep learning models.

#### 4.1.2. Experimental Results and Analysis

This experiment used the vibration signals from the aforementioned dataset. A total of 1024 data points were extracted from the X-direction vibration signals of each sample as input data. The data collected at 20 Hz were selected for noise augmentation and segmentation preprocessing to enhance model robustness. The dataset (500 samples per fault state) was partitioned into training (400 samples) and testing (100 samples) sets in an 8:2 ratio for each health state. The MDSC-SE-BiGRU architecture processes these normalized vibration signals through multiscale depthwise convolution, bidirectional temporal modeling, and channelwise attention mechanisms.

To evaluate the effectiveness of the MDSC-SE-BiGRU model, comparative experiments were conducted using eight different models: (a) 1DCNN-SE (combining one-dimensional CNN and SE attention), (b) 2DCNN-SE (combining two-dimensional CNN and SE attention), (c) 1DCNN-SE-LSTM(combining one-dimensional CNN, SE attention, and LSTM), (d) 2DCNN-SE-LSTM (combining two-dimensional CNN, SE attention, and LSTM), (e) 1DCNN-SE-GRU (combining one-dimensional CNN, SE attention, and GRU), (f) 2DCNN-SE-GRU (combining two-dimensional CNN, SE attention, and GRU), (g) MDSC-ECA-BiGRU(combining the multiscale depthwise separable convolution, Efficient Channel Attention, and BiGRU), and (h)the proposed MDSC-SE-BiGRU model in this study.

The specific parameter settings for the above eight models are detailed in Table 2. Throughout the training process, the iteration cycle was set to 100, the learning rate to 0.001, the batch size to 128, and the stride to 1. All eight models used the ReLU activation function, with the AdamW optimizer and global average pooling.

**Figure 7:**
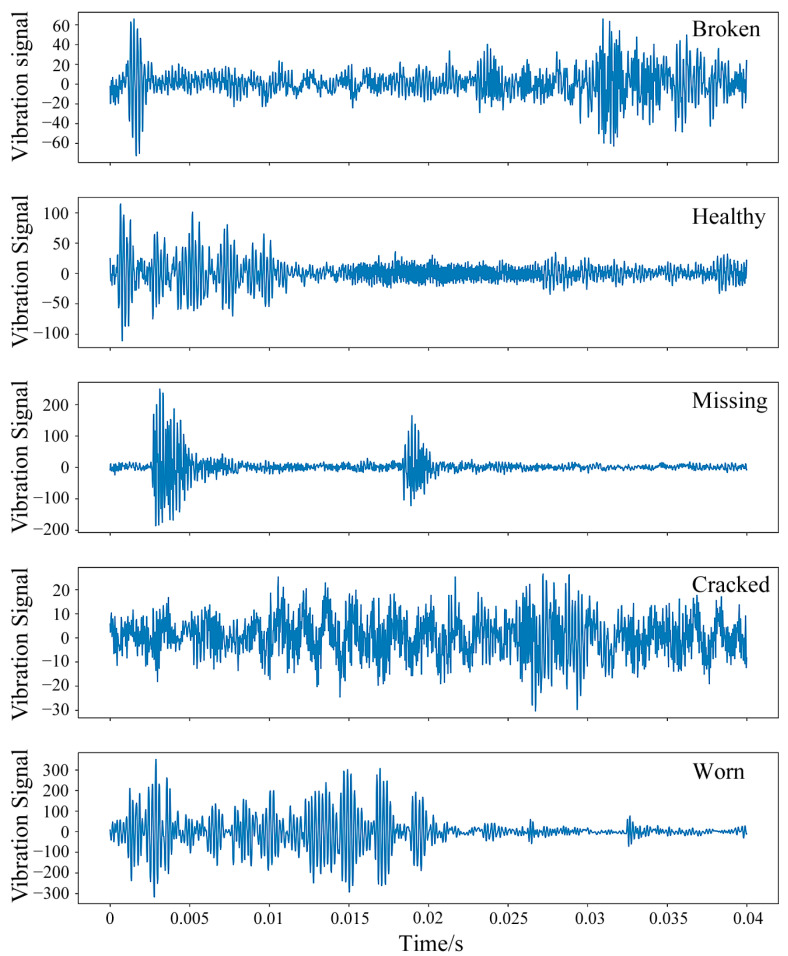
Time-domain distribution of raw data under five different health states in Case 1.

Figure 7 presents the time-domain distributions of raw vibration signals under five different health states at 20 Hz.

**Figure 8:**
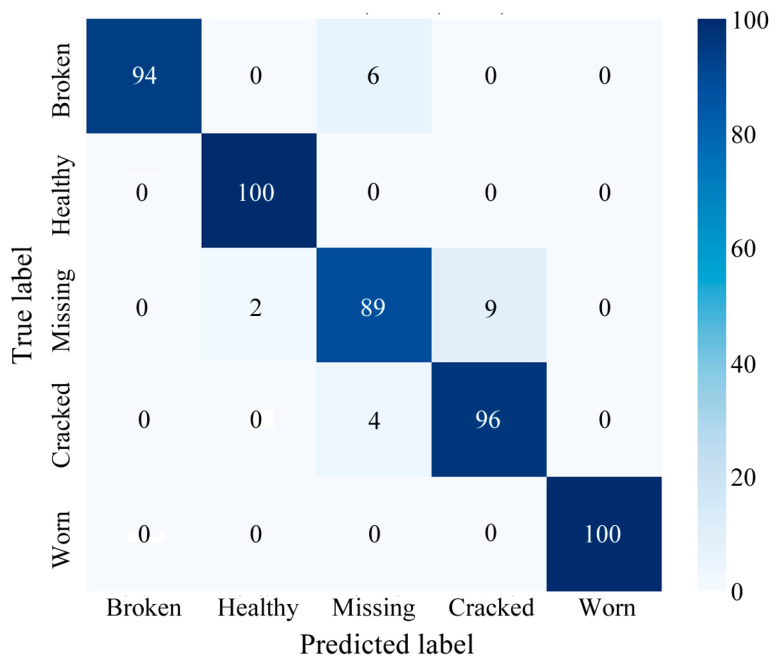
Confusion matrix of MDSC-SE-BiGRU model for fault classification in Case 1.

To validate the effectiveness of the proposed model, a comprehensive evaluation was conducted using confusion matrices and multiple performance metrics. Figure 8 presents the confusion matrix for the MDSC-SE-BiGRU model in gearbox fault classification. Due to inter-class feature similarities across different health conditions and the presence of transient noise interference, some misclassifications were observed in the confusion matrix.

As shown in Table 3, the MDSC-SE-BiGRU model achieved optimal diagnostic performance with an accuracy of 98.80%, surpassing seven baseline models by 1.80–24.80%. This superiority is driven by three key innovations: multiscale spatial feature extraction, dynamic channel optimization, and bidirectional temporal modeling. Compared with 1DCNN-SE, it enhances feature extraction ability through multiscale depthwise separable convolution. In contrast to 2DCNN-SE, the MDSC-SE-BiGRU model addressed inadequate temporal dependency modeling through bidirectional GRU-enhanced contextual learning while surpassing LSTM-based architectures (1DCNN-SE-LSTM; 2DCNN-SE-LSTM) and GRU-based models (1DCNN-SE-GRU; 2DCNN-SE-GRU). Compared to MDSC-ECA-BiGRU, the proposed model achieved a 2.00% accuracy improvement by replacing ECA’s fixed attention with SE’s adaptive channel recalibration, dynamically prioritizing fault-sensitive features. In terms of generalization ability, the MDSC-SE-BiGRU model’s multi-dimensional and sequential feature extraction mechanisms enable it to adapt well to different fault scenarios. The high recall value (98.80%) indicates that the model can effectively identify most of the actual fault cases, demonstrating its robustness and stability in fault diagnosis tasks.

**Figure 9:**
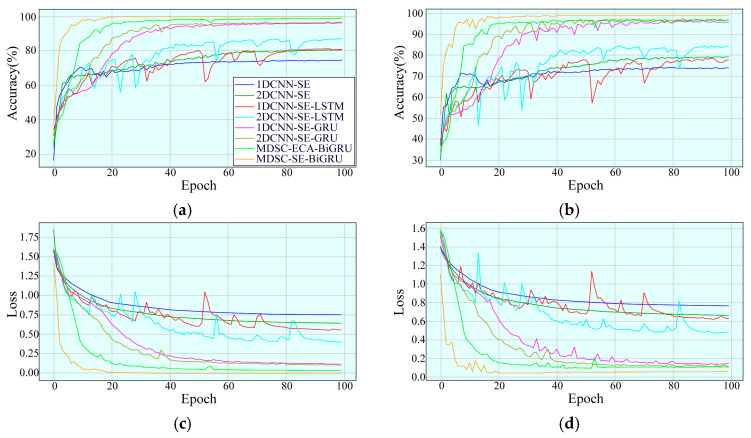
Accuracy and loss curves of eight models in Case 1: (**a**) training accuracy; (**b**) testing accuracy; (**c**) training loss; (**d**) testing loss.

The convergence characteristics of the MDSC-SE-BiGRU model were rigorously assessed by comparing the accuracy and loss trajectories of eight different methods at a rotational speed of 20 Hz in Figure 9. The MDSC-SE-BiGRU model exhibited a notably faster convergence during the early training phase, achieving superior accuracy with minimal fluctuations in the later stages. Additionally, its loss declined sharply during initial iterations and quickly reached a stable state. In contrast, the 1DCNN-SE, 2DCNN-SE, 1DCNN-SE-LSTM, 2DCNN-SE-LSTM, 1DCNN-SE-GRU, 2DCNN-SE-GRU, and MDSC-ECA-BiGRU models demonstrated a slow upward trend in accuracy during the early convergence phase and displayed noticeable fluctuations with lower accuracy compared to the MDSC-SE-BiGRU model in later iterations. Furthermore, they exhibited fluctuating loss trends and ultimately reached higher loss values than the MDSC-SE-BiGRU model. The experimental results confirm that the MDSC-SE-BiGRU model performs more effectively in identifying mechanical faults from complex vibration signals compared to conventional methods.

**Figure 10:**
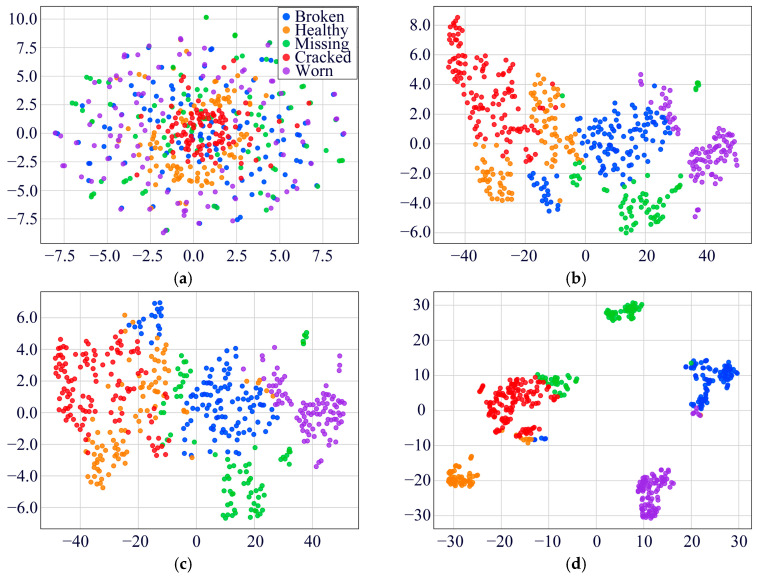

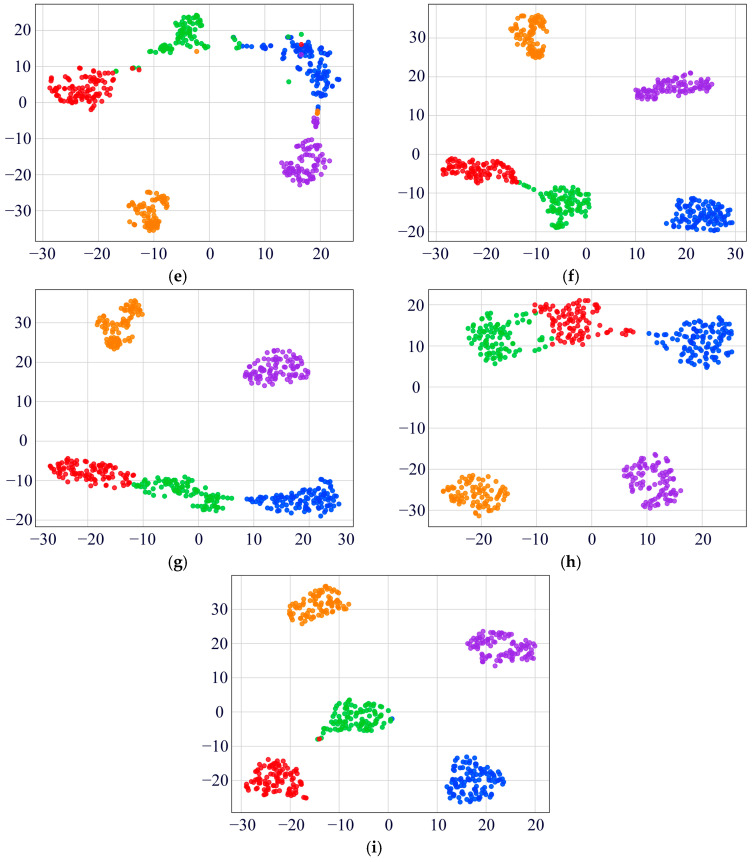
Feature distribution visualization of eight models in Case 1: (**a**) original feature distribution; (**b**) 1DCNN-SE; (**c**) 2DCNN-SE; (**d**) 1DCNN-SE-LSTM; (**e**) 2DCNN-SE-LSTM; (**f**) 1DCNN-SE-GRU; (**g**) 2DCNN-SE-GRU; (**h**) MDSC-ECA-BiGRU; (**i**) MDSC-SE-BiGRU.

To substantiate the diagnostic efficacy of the MDSC-SE-BiGRU model, we conducted a t-SNE visualization of high-dimensional features in Figure 10. The raw vibration signals displayed substantial inter-class overlap, complicating the classification task. Post-training analyses reveal that our model has achieved optimal feature separability, forming distinct clustering patterns for all five fault categories with minimal intra-class dispersion. This indicates that the MDSC-SE-BiGRU model demonstrates superior feature separation in vibration signal classification, thereby validating its practical effectiveness in industrial fault diagnosis.

#### 4.1.3. Testing Performance Under Different Noise Intensities

**Figure 11:**
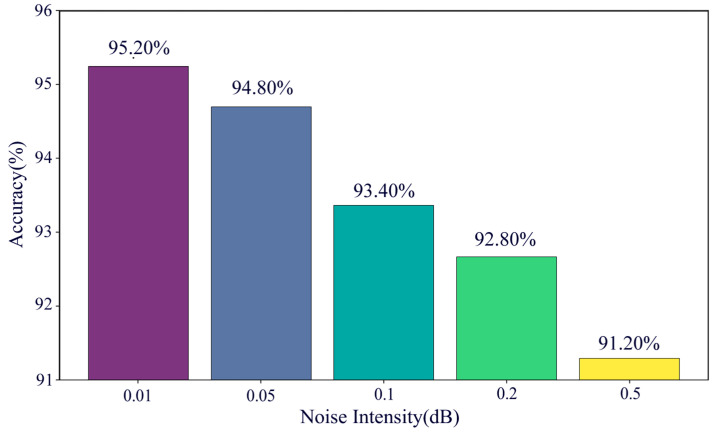
Accuracy of MDSC-SE-BiGRU model under different noise intensities.

To comprehensively assess the robustness of the proposed model under varying noise conditions, this study designed a series of experiments by introducing different noise intensities to simulate the signal interference commonly encountered in real-world industrial scenarios. In the experiment, the noise intensity was progressively set to 0.01, 0.05, 0.1, 0.2, and 0.5, corresponding to a gradual decrease in the Signal–Noise Ratio (SNR). To ensure the comparability of the experimental results, all the tests were conducted using the MDSC-SE-BiGRU model under the same training strategy. The experimental results are illustrated in Figure 11.

The results indicate that as the noise intensity increases, the model’s testing accuracy gradually declines. When the noise intensity was 0.01, the model achieved the highest testing accuracy. This suggests that the model can effectively extract key features from vibration signals and perform accurate fault classification in low-noise environments. However, as the noise intensity increased to 0.5, the testing accuracy significantly decreased, reflecting the adverse impact of high noise levels on model performance. Despite this, the model maintained high classification accuracy above 90% across all the noise levels, demonstrating its robustness and effectiveness in complex noisy environments.

### 4.2. Case 2: Fault Diagnosis of Standard Gearbox

#### 4.2.1. Data Description

**Figure 12:**
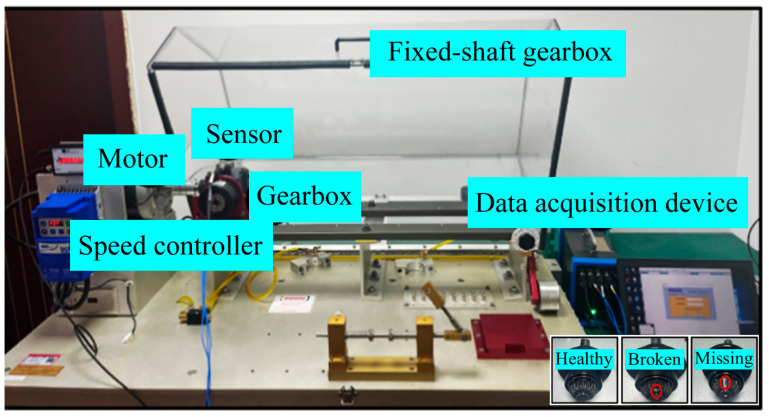
Test bench for Case 2.

Case 2 utilizes the HUST Gearbox Dataset [41]. The data acquisition platform is illustrated in Figure 12. The dataset includes vibration signals collected from the gearbox under three health states (healthy, broken tooth, and missing tooth) and thirty operating conditions (five load types and six rotational speeds).

#### 4.2.2. Experimental Results and Analysis

Each experimental sample comprises 1024 data points acquired from X-axis sensor vibration signals. The operating condition was set at a rotational speed of 40 Hz and a load of 0 N·m for all the fault states. A total of 500 samples were allocated for each fault category, which were divided into 400 training samples and 100 testing samples using an 8:2 partitioning strategy.

**Figure 13:**
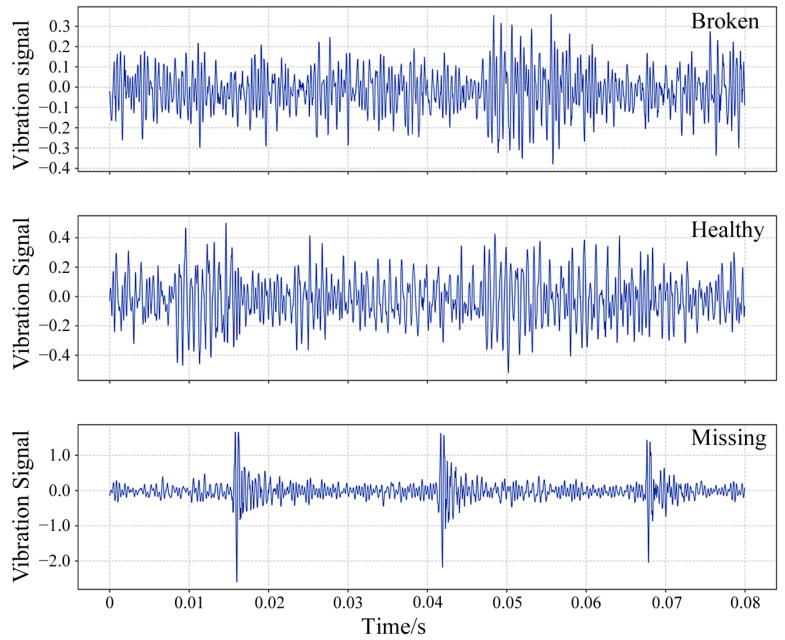
Time-domain distribution of raw data under three different health states in Case 2.

Figure 13 shows the time-domain distribution of raw data across three different health states, gathered under a rotational speed of 40 Hz and a load of 0 N·m.

**Figure 14:**
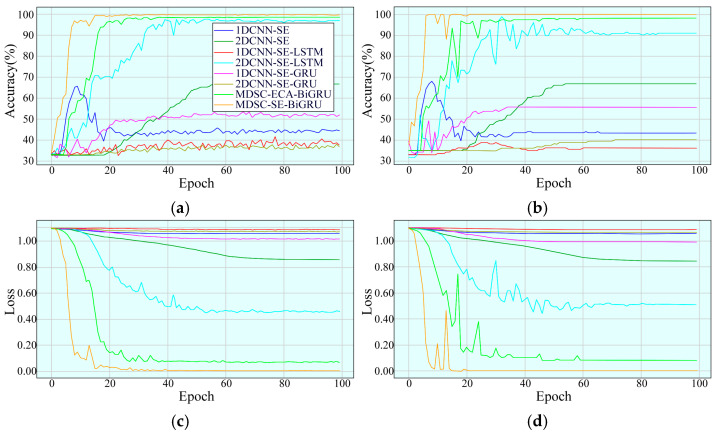
Accuracy and loss curves of eight models in Case 2: (**a**) training accuracy; (**b**) testing accuracy; (**c**) training loss; (**d**) testing loss.

To evaluate the convergence performance of the MDSC-SE-BiGRU model, the accuracy and loss curves of the training and testing sets were analyzed. Figure 14 compares four types of curves for eight diagnostic methods under 40 Hz. From the accuracy curves in Figure 14a,b, it is evident that although MDSC-SE-BiGRU exhibits transient oscillations during the initial training phases, it achieves the highest precision earliest and remains stable throughout. In contrast, 1DCNN-SE, 2DCNN-SE, 1DCNN-SE-LSTM, 1DCNN-SE-GRU, and 2DCNN-SE-GRU models show poor performance throughout the iterations, with negligible accuracy improvements. The accuracy of 2DCNN-SE-LSTM and MDSC-ECA-BiGRU, despite showing an overall upward trend, exhibits considerable fluctuations during the training and testing process, and their final accuracies remain substantially lower than that of MDSC-SE-BiGRU. Compared to 1DCNN-SE, 2DCNN-SE, 1DCNN-SE-LSTM, 2DCNN-SE-LSTM, 1DCNN-SE-GRU, 2DCNN-SE-GRU, and MDSC-ECA-BiGRU, the MDSC-SE-BiGRU model achieves accuracy improvements of 56.49%, 33.12%, 63.64%, 9.09%, 44.16%, 59.74%, and 1.95%, respectively, demonstrating superior accuracy in complex vibration signal classification tasks. From the loss curves in Figure 14c,d, it is evident that the loss value of the MDSC-SE-BiGRU model rapidly decreases during early iterations and eventually stabilizes. In contrast, the loss values of all the other models remain higher than that of MDSC-SE-BiGRU, further confirming its superior loss control capability.

As evidenced in Table 4, the proposed MDSC-SE-BiGRU model demonstrates exceptional diagnostic capabilities, achieving perfect scores across all the metrics and significantly outperforming the seven comparative models. The 1DCNN-SE suffers from inadequate spatial feature representation, while the 2DCNN-SE’s static channel weighting fails to capture temporal dependencies. Although 2DCNN-SE-LSTM achieves moderate performance, its LSTM-based sequential processing is less efficient than our BiGRU design in modeling long-range temporal interactions. Notably, GRU-enhanced variants (1DCNN-SE-GRU, 2DCNN-SE-GRU) underperform due to unidirectional processing and insufficient feature hierarchy, highlighting the necessity of our bidirectional architecture and multiscale feature integration. The 1.95% accuracy improvement of our model over MDSC-ECA-BiGRU validates the superiority of SE’s dynamic channel attention over ECA’s fixed-scale attention, enabling context-aware amplification of fault-related features. The diagnostic performance of MDSC-SE-BiGRU achieves 100.00% in accuracy, precision, recall, and F1 score, achieving precise fault discrimination across all instances and demonstrating exceptional generalization capability to diverse failure modes. The perfect F1 score further confirms a balanced precision–recall of the model, surpassing the conventional models. The proposed model achieves superior feature extraction through the spatiotemporal integration of multiscale depthwise separable feature extraction, adaptive channel recalibration, and bidirectional context-aware temporal modeling—highlighting its remarkable capability in gearbox fault diagnosis and offering a robust foundation for advanced industrial fault detection.

**Figure 15:**
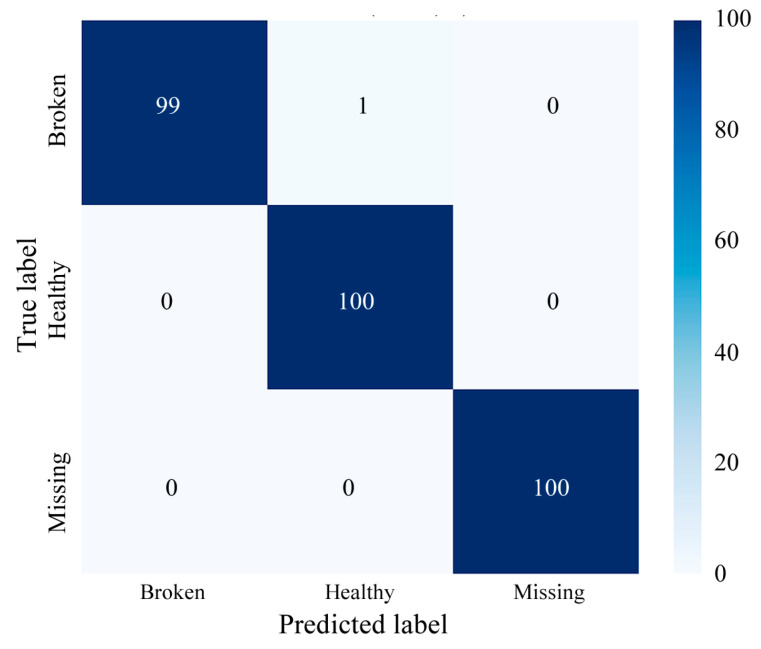
Confusion matrix of MDSC-SE-BiGRU model for fault classification in Case 2.

Figure 15 presents the confusion matrix for the MDSC-SE-BiGRU model in gearbox fault diagnosis.

**Figure 16:**
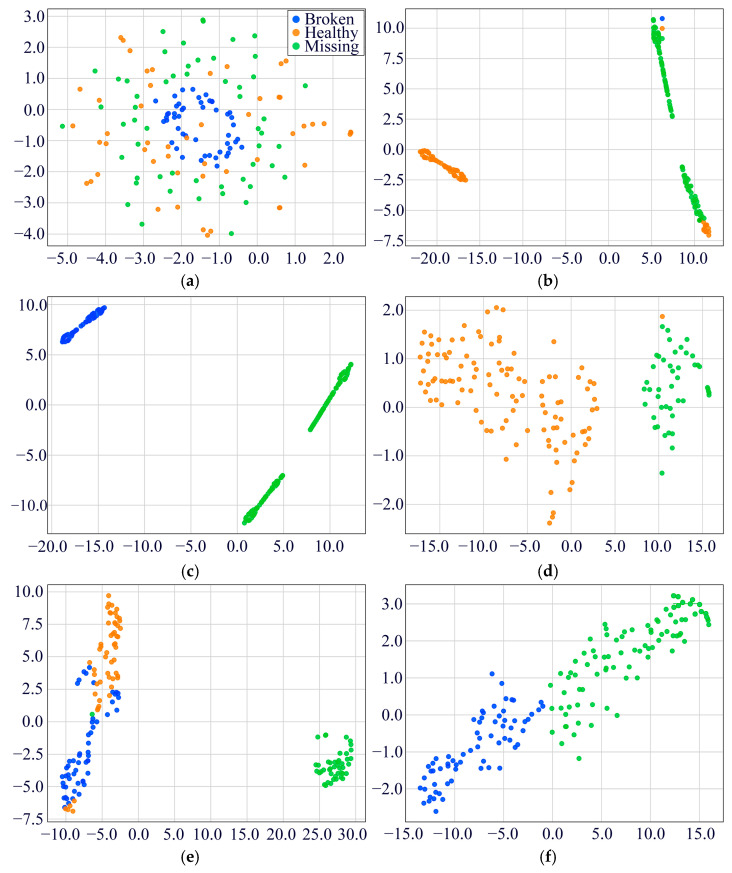

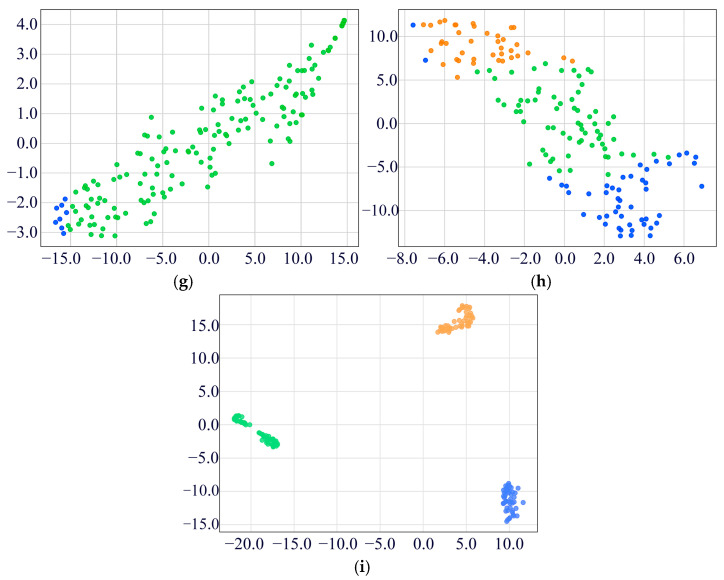
Feature distribution visualization of the eight models in Case 2: (**a**) original feature distribution; (**b**) 1DCNN-SE; (**c**) 2DCNN-SE; (**d**) 1DCNN-SE-LSTM; (**e**) 2DCNN-SE-LSTM; (**f**) 1DCNN-SE-GRU; (**g**) 2DCNN-SE-GRU; (**h**) MDSC-ECA-BiGRU; (**i**) MDSC-SE-BiGRU.

To verify the classification ability of the MDSC-SE-BiGRU model, we used t-SNE technology to visualize and analyze the feature distribution. Figure 16 compares the processed results of the original test data with those from eight diagnostic methods. The unprocessed original features (Figure 16a) exhibit obvious category overlap among distinct fault types; Figure 16b–d,f,g reveal the absence of a specific class in the t-SNE visualizations of certain models, indicating insufficient feature separability for that class in high-dimensional space. This deficiency directly reflects limitations in the model’s feature extraction capability, where crucial features of the missing class are inadequately captured. As a result, the features overlap with other classes after dimensionality reduction, rendering the class invisible in low-dimensional projections. In comparison with the other seven fault diagnosis methods, the features processed by MDSC-SE-BiGRU (Figure 16i) show the clearest category boundaries, with tightly clustered features of the same type and significantly separated features of different types. This comparison confirms the superiority of the model in classifying complex vibration signals.

#### 4.2.3. Testing Performance Under Different Operating Conditions

**Figure 17:**
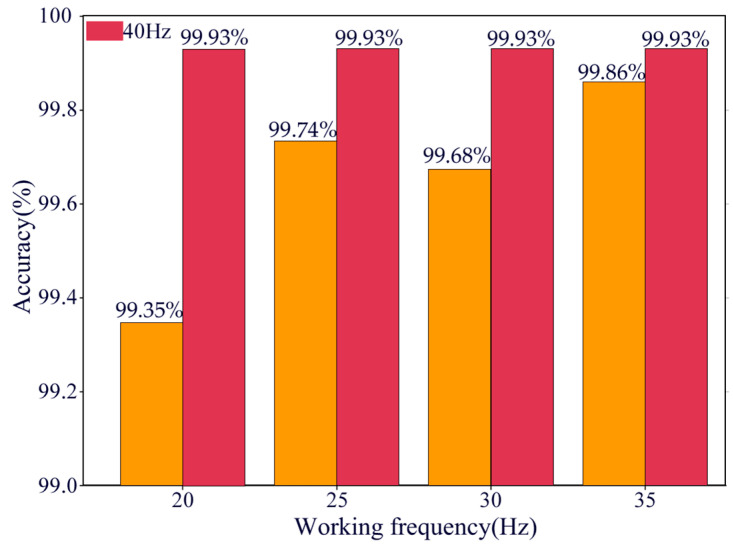
Accuracy of MDSC-SE-BiGRU model under different working conditions.

To comprehensively evaluate the robustness and generalization ability of the proposed model under varying operating conditions, experiments were conducted at five different rotational speeds: 20 Hz, 25 Hz, 30 Hz, 35 Hz, and 40 Hz. The model’s performance was assessed using key evaluation metrics, including training and testing accuracy, loss variation curves, and t-SNE visualizations. The experimental results demonstrate that the model maintains high classification accuracy across all the operating conditions, with notable improvements in both accuracy and stability—especially at higher speeds. The fault diagnosis accuracy under different operating conditions is illustrated in Figure 17.

**Figure 18:**
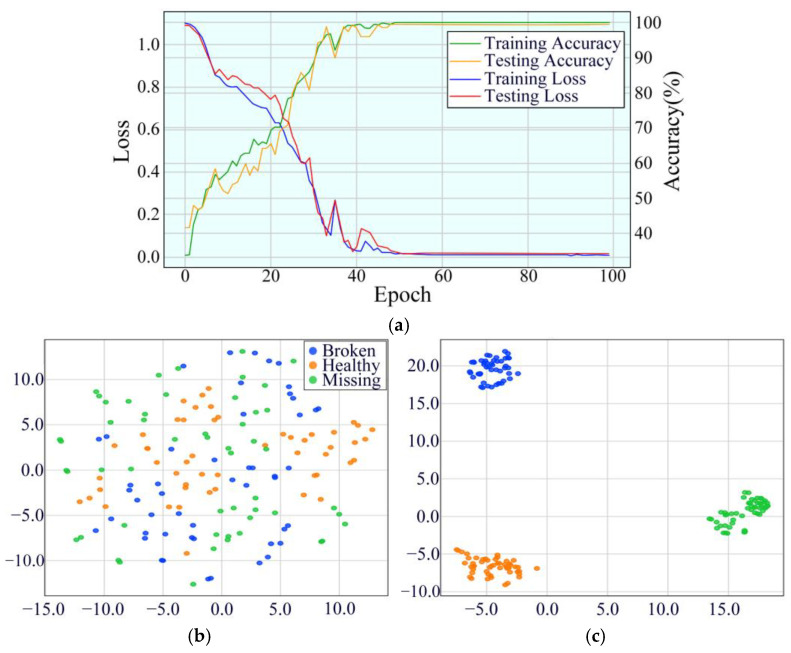
Performance evaluation indicators of MDSC-SE-BiGRU model at 20 Hz: (**a**) accuracy and loss curves; (**b**) t-SNE before classification; (**c**) t-SNE after classification.

At 20 Hz, the model’s initial training accuracy was 33.71%, and the testing accuracy was 41.56%. As training progressed, the model’s accuracy steadily improved, ultimately reaching 99.84% training accuracy and 99.35% testing accuracy by the 100th epoch. Despite some fluctuations in the early training stages, the model demonstrated strong stability in later stages, particularly after the 32nd epoch, where the testing accuracy remained consistently above 90%. This indicates that the model can effectively extract key features from vibration signals and perform accurate fault classification under low-speed conditions. The model’s performance is demonstrated in Figure 18.

**Figure 19:**
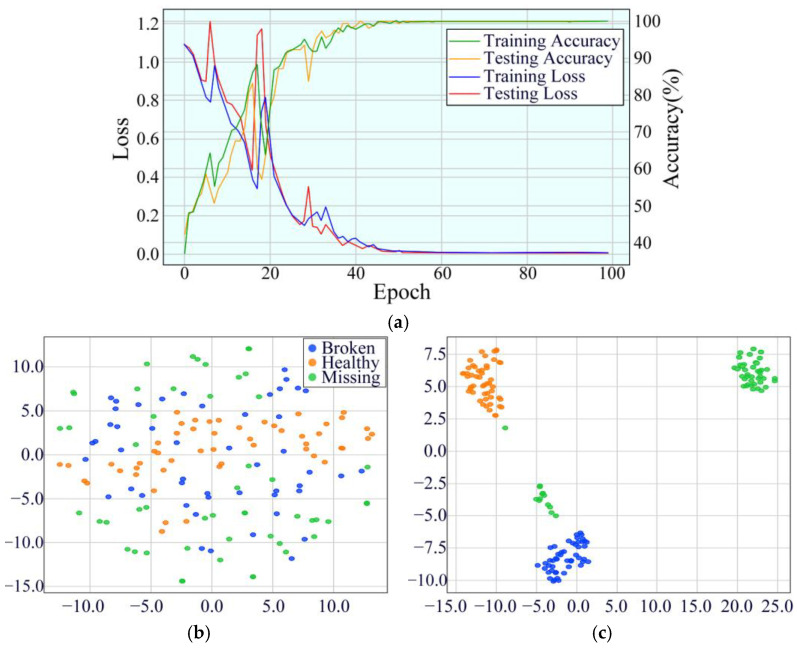
Performance evaluation indicators of MDSC-SE-BiGRU model at 25 Hz: (**a**) accuracy and loss curves; (**b**) t-SNE before classification; (**c**) t-SNE after classification.

At 25 Hz, the model’s initial training accuracy was 37.13%, and the testing accuracy was 42.21%. With continuous training, the accuracy improved significantly, ultimately reaching 99.95% training accuracy and 99.74% testing accuracy at the 100th epoch. Compared to 20 Hz, the model exhibited a faster convergence speed and achieved higher stability during high-iteration cycles under 25 Hz conditions. This suggests that the model possesses stronger generalization capability under medium-speed conditions. The model’s performance is displayed in Figure 19.

**Figure 20:**
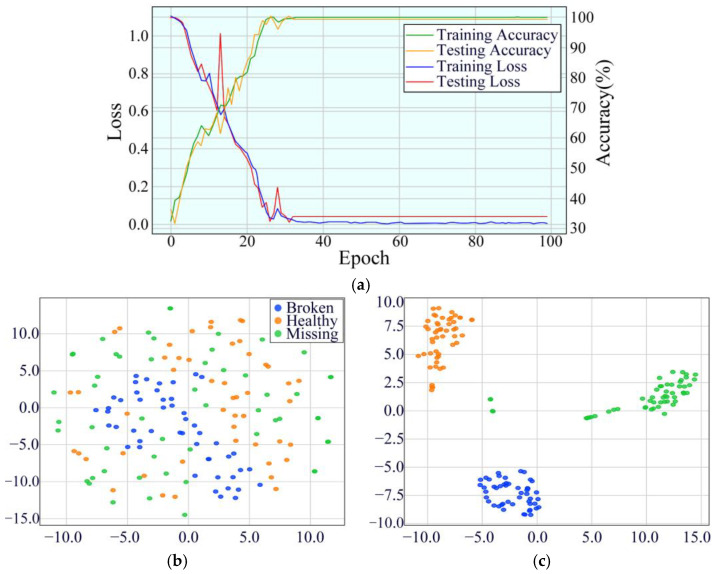
Performance evaluation indicators of MDSC-SE-BiGRU model at 30 Hz: (**a**) accuracy and loss curves; (**b**) t-SNE before classification; (**c**) t-SNE after classification.

At 30 Hz, the model’s initial training accuracy was 34.20%, and the testing accuracy was 27.92%. Despite the relatively low initial accuracy, the model’s performance improved rapidly during training, achieving 99.89% training accuracy and 99.68% testing accuracy at the 100th epoch. Compared to 20 Hz and 25 Hz, the model exhibited greater fluctuations during the early training stages at 30 Hz, but ultimately achieved higher stability in later stages. This indicates that the model can effectively handle complex vibration signals at higher speeds. The model’s performance is showcased in Figure 20.

**Figure 21:**
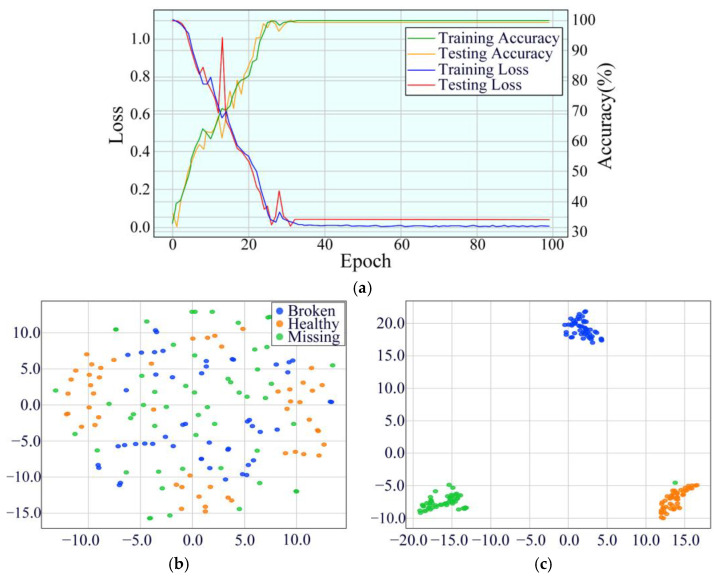
Performance evaluation indicators of MDSC-SE-BiGRU model at 35 Hz: (**a**) accuracy and loss curves; (**b**) t-SNE before classification; (**c**) t-SNE after classification.

At 35 Hz, the model’s initial training accuracy was 32.74%, and the testing accuracy was 36.36%. With ongoing training, both accuracies improved progressively, ultimately reaching 99.86% for both training and testing accuracy at the 100th epoch. Compared to 30 Hz, the model exhibited less fluctuation in the early training phase and achieved higher stability during high-iteration periods under 35 Hz conditions. This suggests that the model can maintain strong classification performance at even higher rotational speeds. The model’s performance is depicted in Figure 21.

**Figure 22:**
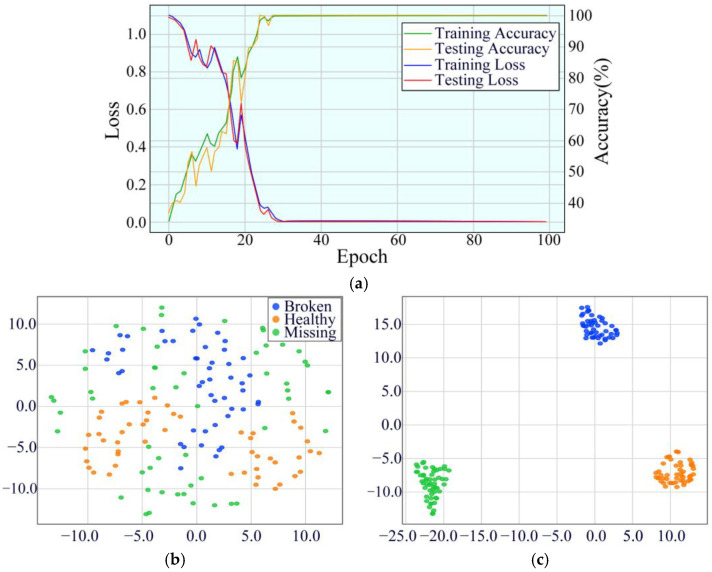
Performance evaluation indicators of MDSC-SE-BiGRU model at 40 Hz: (**a**) accuracy and loss curves; (**b**) t-SNE before classification; (**c**) t-SNE after classification.

At 40 Hz, the model’s initial training accuracy was 34.04%, and the testing accuracy was 37.01%. As training progressed, the model achieved 99.93% training and testing accuracy at the 100th epoch. Compared to 35 Hz, the model exhibited even greater stability during high-iteration periods under 40 Hz conditions. This indicates that the model can consistently maintain excellent classification performance even under extremely high-speed conditions. The model’s performance is presented in Figure 22.

A comprehensive analysis of the results across five different operating conditions reveals that the model demonstrates high classification performance and accuracy across varying rotational speeds. As the speed increases, the convergence speed and stability of the model gradually improve. Especially under high-speed conditions, the accuracy and stability of the model are significantly enhanced. These findings confirm that the model exhibits strong robustness and exceptional generalization capability under diverse operating conditions, effectively handling complex vibration signals and providing reliable technical support for intelligent gearbox fault diagnosis.

### 4.3. Ablation Experiments for the Proposed MDSC-SE-BiGRU Model

To systematically evaluate the contributions of the multiscale feature extraction module (MDSC), attention mechanism module (SE), and bidirectional gated recurrent unit module (BiGRU) in the proposed MDSC-SE-BiGRU framework, we conducted ablation studies to validate the effectiveness of their integration. As detailed in Table 5, the experimental parameters were kept consistent across all the configurations, while the same two datasets described in previous sections were used. The final results were obtained by averaging the outcomes of two independent trials to ensure statistical reliability.

Experiment 1, using only 1DCNN, exhibits poor diagnostic ability due to its limited ability to capture multiscale vibration features. Introducing MDSC in Experiment 2 results in a noticeable upward trend across all the metrics, underscoring the effectiveness of parallel multiscale feature extraction in capturing fault signatures distributed across varying frequency components. When SE is individually incorporated in Experiment 3, moderate gains are observed, attributed to its ability to adaptively recalibrate channelwise features, thus enhancing informative components and suppressing irrelevant patterns. Experiment 4, which solely employs BiGRU, demonstrates a significant increase in performance, indicating the critical role of bidirectional temporal modeling in capturing non-stationary and sequential characteristics of vibration signals. The integration of SE with BiGRU in Experiment 5 achieves enhanced temporal modeling, where SE’s channelwise attention mechanism adaptively highlights critical fault-related features, enabling BiGRU to more effectively capture their temporal evolution while suppressing noise-induced variations. When MDSC is paired with BiGRU in Experiment 6, the performance of the algorithm continues to ascend, illustrating the strong spatiotemporal synergy between multiscale convolution and bidirectional time-series modeling. In contrast, Experiment 7, which integrates MDSC and SE without BiGRU, fails to sustain the performance gains achieved when either module is paired with temporal modeling. This indicates that spatial and channelwise feature enhancements alone are insufficient to capture the temporal dependencies critical for accurate fault characterization, highlighting the essential role of sequence modeling in vibration-based diagnostics. The synergistic integration of MDSC, SE, and BiGRU in Experiment 8 culminates in near-flawless diagnostic performance, which demonstrates that the coordinated fusion of multiscale spatial extraction, adaptive feature recalibration, and bidirectional temporal modeling enables the model to better capture fault signatures.

Beyond the validation of the experimental results, the proposed architecture is also supported by a strong theoretical foundation. The MDSC module is specifically designed to capture the multiscale characteristics inherent in vibration signals caused by compound and interacting faults. By employing dilated convolutions, it effectively expands the receptive field without increasing computational complexity, allowing the model to extract both localized transient features and broader frequency patterns. The SE module enhances fault-sensitive frequency components while suppressing irrelevant or noisy information, guided by the energy distribution of the signal. The BiGRU module is incorporated to capture temporal dependencies in the signal, especially under variable-speed conditions where fault signatures change over time. Unlike feedforward networks, BiGRU leverages bidirectional context, enabling it to learn both past and future dependencies, which are critical for tracking fault progression. This model combines the advantages of the three modules mentioned above to effectively extract key fault features and perform accurate feature classification, thereby achieving both theoretical rationality and practical effectiveness.

## 5. Conclusions

This study innovatively proposes an intelligent fault diagnosis model for gearboxes, namely MDSC-SE-BiGRU. This model introduces multiscale feature extraction (MDSC module, which effectively captures hierarchical features across different scales), adaptive feature enhancement (SE module, which dynamically adjusts feature weights), and bidirectional time modeling (BiGRU module, which accurately learns sequence dependencies), significantly improving the extraction and classification of key fault features. To verify the generalization ability of the model, two independent gearbox fault datasets were divided into a training set and a testing set using an 8:2 split under identical model architectures and parameter settings. In Case 1, the model achieved an accuracy of 98.80%, while in Case 2, the accuracy reached 100.00%. The results indicate that the proposed model performs well across both datasets. Under the same experimental conditions, all the performance indicators of this model were superior to those of the comparison models. Moreover, even under noise and variable operating conditions, the model maintained strong robustness and generalization ability.

Key experimental evidence confirms that the proposed MDSC-SE-BiGRU has the following core advantages: (1) The MDSC module captures local and global feature information through multiscale feature fusion and decomposes standard convolution into depthwise and pointwise convolutions, reducing the number of parameters. This addresses low computational efficiency due to insufficient scale adaptability, limited contextual information, and receptive field redundancy in 1DCNN and 2DCNN. (2) The SE module dynamically prioritizes key fault features by learning the importance of each channel. It achieved an accuracy that was 1.95% higher than fixed-attention ECA, validating its effectiveness in attention weighting. (3) The bidirectional temporal modeling of BiGRU solves the unidirectional limitations of LSTM and GRU, improving context understanding. Compared with 2DCNN-SE-LSTM, the F1 score increased by 9.13%.

Based on the findings and limitations of this study, future research can focus on the following directions:

(1) Acquiring real-world industrial fault data to further validate the performance of the MDSC-SE-BiGRU model in practical applications.
(2) Optimizing the computational efficiency of the MDSC-SE-BiGRU model to meet the real-time diagnostic requirements of industrial applications, enabling lightweight deployment on embedded systems and edge computing platforms.
(3) Integrating vibration, temperature, acoustic, and other multi-modal signals to further improve the accuracy and reliability of fault diagnosis.

In summary, the proposed MDSC-SE-BiGRU model offers an efficient and reliable solution for intelligent gearbox fault diagnosis. Future research efforts will further support its deployment and widespread adoption in real-world industrial applications.

## Figures and Tables

**Table 1:** Network parameters for MDSC-SE-BiGRU.

Layer Structure	Input Size	Convolutional Kernel Size (Number)	Output Size	Stride
Conv_1-BN-ReLU	[1, 1024]	3 × 1 (64)	[64, 1024]	1
Conv_2-BN-ReLU	[1, 1024]	5 × 1 (64)	[64, 1024]	1
Conv_3-BN-ReLU	[1, 1024]	7 × 1 (64)	[64, 1024]	1
Feature Fusion	[64, 1024] × 3	-	[192, 1024]	-
SEBlock	[192, 1024]	-	[192, 1024]	-
BiGRU	[192, 1024]	-	[256, 1024]	-
Fc_1	[256]	-	[128]	-
Fc_2	[128]	-	[64]	-
Fc_3	[64]	-	[5]	-

**Table 2:** Parameter list of eight fault diagnosis models.

Model Name	Kernel Size	Input Size	Output Size	Parameter Number	FC Layers
1DCNN-SE	3	(1, 1024)	(Batch, 5)	23,429	2
2DCNN-SE	5	(1, 1024)	(Batch, 5)	23,429	2
1DCNN-SE-LSTM	3	(1, 1024)	(Batch, 5)	173,445	2
2DCNN-SE-LSTM	5	(1, 1024)	(Batch, 5)	173,445	2
1DCNN-SE-GRU	3	(1, 1024)	(Batch, 5)	139,397	2
2DCNN-SE-GRU	5	(1, 1024)	(Batch, 5)	139,397	2
MDSC-ECA-BiGRU	3, 5, 7	(1, 1024)	(Batch, 5)	564,933	3
MDSC-SE-BiGRU	3, 5, 7	(1, 1024)	(Batch, 5)	564,421	3

**Table 3:** Evaluating indicators of eight fault diagnosis models.

Model Name	Accuracy	Precision	Recall	F1 Score
1DCNN-SE	0.7400	0.7574	0.7400	0.7423
2DCNN-SE	0.7920	0.7962	0.7920	0.7912
1DCNN-SE-LSTM	0.7780	0.7987	0.7780	0.7796
2DCNN-SE-LSTM	0.8400	0.8406	0.8400	0.8395
1DCNN-SE-GRU	0.9580	0.9587	0.9580	0.9582
2DCNN-SE-GRU	0.9700	0.9700	0.9700	0.9700
MDSC-ECA-BiGRU	0.9680	0.9682	0.9680	0.9680
MDSC-SE-BiGRU	0.9880	0.9880	0.9880	0.9880

**Table 4:** Evaluation indicators of eight fault diagnosis models.

Model Name	Accuracy	Precision	Recall	F1 Score
1DCNN-SE	0.4351	0.2753	0.4351	0.4351
2DCNN-SE	0.6688	0.4985	0.6688	0.5564
1DCNN-SE-LSTM	0.3636	0.2876	0.3636	0.3034
2DCNN-SE-LSTM	0.9091	0.9142	0.9091	0.9087
1DCNN-SE-GRU	0.5584	0.5584	0.5584	0.4493
2DCNN-SE-GRU	0.4026	0.4479	0.4026	0.2787
MDSC-ECA-BiGRU	0.9805	0.9682	0.9805	0.9805
MDSC-SE-BiGRU	1.0000	1.0000	1.0000	1.0000

**Table 5:** Results of ablation experiment.

Number	MDSC	SE	BiGRU	Accuracy	Precision	Recall	F1 Score
1	×	×	×	0.4720	0.5408	0.4720	0.4741
2	√	×	×	0.6040	0.6233	0.6040	0.6108
3	×	√	×	0.5660	0.5916	0.5660	0.5627
4	×	×	√	0.7340	0.7345	0.7340	0.7331
5	×	√	√	0.8940	0.8952	0.8940	0.8916
6	√	×	√	0.9100	0.9102	0.9100	0.9098
7	√	√	×	0.8200	0.8219	0.8200	0.8193
8	√	√	√	0.9940	0.9940	0.9940	0.9940

Notes: √ indicates the inclusion of a module; × indicates the removal of a module.

## Data Availability

The data presented in this study are available in [WT-Planetary gearbox dataset] at [https://github.com/Liudd-BJUT/WT-planetary-gearbox-dataset] (accessed on 1 February 2025), reference number [40], and in [HUST Gearbox Dataset] at [https://github.com/CHAOZHAO-1/HUSTgearbox-dataset] (accessed on 1 February 2025), reference number [41].
